# Stability and Activity of Rhodium Promoted Nickel-Based Catalysts in Dry Reforming of Methane

**DOI:** 10.3390/nano13030547

**Published:** 2023-01-29

**Authors:** Jehad Saleh, Ahmed Sadeq Al-Fatesh, Ahmed Aidid Ibrahim, Francesco Frusteri, Ahmed Elhag Abasaeed, Anis Hamza Fakeeha, Fahad Albaqi, Khalid Anojaidi, Salwa B. Alreshaidan, Ibrahim Albinali, Abdulrahman A. Al-Rabiah, Abdulaziz Bagabas

**Affiliations:** 1 Chemical Engineering Department, College of Engineering, King Saud University, P.O. Box 800, Riyadh 11421, Saudi Arabia; 2 CNR-ITAE, Istituto di Tecnologie Avanzate per Energia “Nicola Giordano”, Via S. Lucia Sopra Contesse 5, 98126 Messina, Italy; 3 President Office, King Abdulaziz City for Science and Technology (KACST), P.O. Box 6086, Riyadh 11442, Saudi Arabia; 4 Department of Chemistry, Faculty of Science, King Saud University, P.O. Box 800, Riyadh 11451, Saudi Arabia

**Keywords:** methane dry reforming, yttria-stabilized zirconia support, nickel-based catalyst, Rh_2_O_3_ promoter, TGA results

## Abstract

The rhodium oxide (Rh_2_O_3_) doping effect on the activity and stability of nickel catalysts supported over yttria-stabilized zirconia was examined in dry reforming of methane (DRM) by using a tubular reactor, operated at 800 °C. The catalysts were characterized by using several techniques including nitrogen physisorption, X-ray diffraction, transmission electron microscopy, H_2_-temperature programmed reduction, CO_2_-temperature programmed Desorption, and temperature gravimetric analysis (TGA). The morphology of Ni-YZr was not affected by the addition of Rh_2_O_3_. However, it facilitated the activation of the catalysts and reduced the catalyst’s surface basicity. The addition of 4.0 wt.% Rh_2_O_3_ gave the optimum conversions of CH_4_ and CO_2_ of ~89% and ~92%, respectively. Furthermore, the incorporation of Rh_2_O_3_, in the range of 0.0–4.0 wt.% loading, enhanced DRM and decreased the impact of reverse water gas shift, as inferred by the thermodynamics analysis. TGA revealed that the addition of Rh_2_O_3_ diminished the carbon formation on the spent catalysts, and hence, boosted the stability, owing to the potential of rhodium for carbon oxidation through gasification reactions. The 4.0 wt.% Rh_2_O_3_ loading gave a 12.5% weight loss of carbon. The TEM images displayed filamentous carbon, confirming the TGA results.

## 1. Introduction

It is anticipated that energy demand in 2030 will be 55% more than that in the last three decades [1]. However, it is essential to have clean energy in the light of the tighten environmental legislations. In this content, the abundant natural gas reserves could provide a solution because of its cleaner combustion and easier reforming than the crude oil [2]. Different reforming techniques are employed for CH_4_ valorisation. For instance, steam reforming of methane (SRM) [3], dry reforming of methane (DRM) [4], and partial oxidation of methane (POM) [5]. The reforming of CH_4_, the major constituent of natural gas, can be performed to produce synthesis gas (syngas, CO and H_2_ mixture) via one of the following three processes:

(1) $${\mathrm{CH}}_{4}+{\mathrm{CO}}_{2}↔2H_{2}+2\mathrm{CO}\;{\Delta H}_{298}^{{}^{\circ}}=+247\frac{\mathrm{kJ}}{\mathrm{mol}}\;\mathrm{dry}\;\mathrm{reforming}$$

(2) $${\mathrm{CH}}_{4}+H_{2}O↔3H_{2}+\mathrm{CO}\;{\Delta H}_{298}^{{}^{\circ}}=+206\frac{\mathrm{kJ}}{\mathrm{mol}}\;\mathrm{steam}\;\mathrm{reforming}$$

(3) $${\mathrm{CH}}_{4}+\frac{1}{2}O_{2}↔2H_{2}+\mathrm{CO}\;{\Delta H}_{298}^{{}^{\circ}}=-36\frac{\mathrm{kJ}}{\mathrm{mol}}\;\mathrm{partial}\;\mathrm{oxidation}$$

These processes have presently revived research interests [6,7,8].

Indeed, dry reforming of methane (DRM), which is an environmentally friendly and energetically striking method utilizes the greenhouse gases CH_4_ and CO_2_ to generate syngas with an equal H_2_/CO mole ratio, which is appropriate for Fischer–Tropsch syngas to produce liquid fuel [9]. Compared with other methane reforming processes, the overall operation cost is estimated to be 20% lower to produce high purity syngas with low CO_2_ content [10]. However, the DRM process has serious drawbacks; the core ones are related to energy costs due to high endothermicity and catalyst deactivation (carbon deposition and sintering), limiting its commercial application [11]. The carbon formation can be resolved from the catalyst by construction modification. To avoid homogeneous cracking and coking of hydrocarbons, highly active metals should be chosen [12,13]. Noble metals such as platinum (Pt), rhodium (Rh), and palladium (Pd) have been widely investigated due to their superior performance, but their high prices restrain their application [14,15]. Cheap transition metals are the potential switch. For instance, nickel (Ni), as an active site, has been studied and shown good performance in DRM with a high syngas selectivity [16]. A catalyst with 5 wt.% nickel oxide loading was employed in long-term stability testing and showed stable catalytic performance up to 50 h time-on-stream without any decrease in methane conversion in the dry reforming process [17]. However, the eminent propensity of Ni to sintering and coking under DRM conditions is the major drawback in the development of stable Ni-based catalysts [18]. The design of cost-effective, efficient DRM catalysts is a grand challenge in this topic. Bimetallic catalysts, providing synergistic effects via metal-to-metal interactions, appear to be an effective strategy for achieving these goals. The addition of a noble metal to Ni-based catalysts characteristically results in a higher dispersion of metal particles, owing to the dilution effect of the noble metal, smaller alloyed particles, enhanced reducibility, and better resistance to oxidation, sintering, and carbon formation, favouring DRM performance and time-on-stream stability [19,20]. For the selection of the active metal, researchers have investigated Rh, Ru, Pt, and Ni. Rh showed the highest activity and best anti-coking performance [21]. The Ni-Rh bimetallic system may be satisfactory for engineering applications when considering the performance and cost [22]. Neuberg et al. highlighted the promoting consequence of Rh on the activity and stability of Pt-based methane combustion catalyst (Pt-Rh/γ-Al_2_O_3_) [23]. The Rh-promoted catalysts showed better stability, as a result of developing smaller crystal sizes. Alternatively, Arbag et al. reported better performance and stability of Ni/MCM-41ctalaytst in DRM due to the reduction of reverse water gas shift reaction [24]. Khalighi et al. examined the catalytic performance of Rh- and Ni-containing catalysts, supported by CoAl_2_O_4_ in DRM. They found that the 3 wt.% Rh-containing catalyst gave the maximum methane conversion with no deactivation or carbon formation [25]. Tarifa et al. studied steam reforming of clean biogas over Rh or Ru catalysts supported on NiCrAl open-cell metallic foam [26]. The Rh catalysts showed great activity, stability, and low carbon deposition due to the presence of large Ni particles. Ocsachoque et al. investigated the activity, stability, and carbon deposition of a Rh-promoted Ni-based catalyst (Ni/Al_2_O_3_) during DRM [1]. Their outcome displayed that the Rh addition favoured metal-support interaction and provided a high activity. Furthermore, the elimination of carbon deposition can be enhanced by selecting a support with good oxygen storage/release abilities and less acidic sites [27]. ZrO_2_ has good thermal stability and three crystal structures (monoclinic, tetragonal, and cubic) [28]. The various ZrO_2_ forms also have different porous structures, which might influence the properties of the generated coke. ZrO_2_, as a support, exhibited better stability during calcination and reforming due to a high Hüttig and Tamman temperature [11]. Zhang et al. [29] used ZrO_2_ for steam reforming and found that the modified catalysts, like Y_2_Zr_2_O_7_, had a pyrochlore structure with more mobile oxygen species to enhance coking resistance. Support optimization is a good way to prevent carbon deposition. Supports with higher surface basicity showed higher DRM efficiency. Modification of catalyst support helped to improve the catalyst stability with less carbon deposition [30,31]. Pedrero et al. investigated the partial oxidation of methane and ethane over Rh and Ni supported on zirconia-modified alumina catalysts. Their results stipulated that the modified support improved significantly the performances of the catalysts [32]. Similarly, Lv et al. reported the dry reforming of low-carbon alkane over Ni/La_2_O_3_-ZrO_2_. Their results showed high performance and stability, which were due to the modifications of support and the formation of a stable pyrochlore La_2_Zr_2_O_7_ [33]. When Y_2_O_3_ is doped with transition metals such as Zr, the number of oxygen vacancies and thermal stability are further improved. Impregnation is a common catalyst preparation method, which is predominant in the production of industrial catalysts [34]. The addition of yttria into zirconia supports prevents phase transition in zirconia at high reaction temperatures [35]. Moreover, it compensates for the vacancy in zirconia, offers oxygen ion conductivity in the lattice, obstructs the reaction of carbon deposit with ZrO_2_ support, confines the crystalline size of NiO, and improves the surface parameter as well as develops strong basic sites [35,36,37]. Therefore, it is convincing and essential to develop an innovative catalyst with satisfactory physicochemical properties to employ in the DRM process. On this basis, we were motivated to combine Rh and Ni, and supported them on yttria-stabilized zirconia catalysts for DRM. The effect of different loadings of Rh_2_O_3_ on the activity and stability of 5 wt.% NiO at 800 °C, were tested for determining the optimum loading. The current work aims to get a further comprehensive vision of the role of Rh_2_O_3_ on the DRM reaction and establish the coke-resistant effect of this promoter. The effect of Rh_2_O_3_ loading was explored by suitable characterization techniques.

## 2. Materials and Methods

### 2.1. Material

The chemicals used were obtained commercially and were used without further purification. Nickel nitrate hexahydrate [Ni (NO_3_)_2_.6H_2_O] was obtained from Riedel-De Haen AG, Seelze, Germany, rhodium chloride (RhCl_3_) was purchased from Sigma-Aldrich (Ward Hill, MA, USA), and the 8.0 wt.% yttria-stabilized zirconia was purchased from Alfa Aesar (St. louis, Mo, USA).

### 2.2. Catalyst Preparation

The wet impregnation method was employed to prepare the rhodium-promoted, yttria-stabilized zirconia-supported nickel catalysts. The appropriate amounts of nickel nitrate hexahydrate to obtain 5.0 wt.% NiO, rhodium chloride to obtain 1.0, 2.0, 3.0, 4.0, or 5.0 wt.% Rh_2_O_3_, and the support were mixed and ground together. Drops of ultrapure water were added to the solid mixture to make a paste. Subsequently, this paste was stirred mechanically until it was dried. Wetting and drying were performed three times for ensuring the homogeneous distribution of the components within the support matrix. Calcination at 600 °C for three hours was then performed to obtain the rhodium-promoted, yttria-stabilized zirconia-supported nickel catalysts, which were abbreviated as Rh-x (x = 0, 1, 2, 3, 4, 5).

### 2.3. Catalyst Characterization

The catalysts were characterized by N_2_ adsorption–desorption at −196 °C by using a Micromeritics Tristar II 3020 for porosity and surface area analyses. Hydrogen temperature-programmed reduction was performed using 0.070 g of catalyst, placed inside the holder of a Micromeritics AutoChem II apparatus. The readings were taken at 150 °C under argon gas flow for half an hour, followed by cooling to ambient temperature. The next step involved heating by the furnace up to 800 °C, ramping at 10 °C min^−1^ in an atmosphere of a H_2_/Ar mixture (1:9 vol. %), flowing at 40 mL/min. The thermal conductivity unit recorded the H_2_ consumption. The X-ray diffraction patterns of the catalysts were recorded on a Miniflex Rigaku diffractometer, equipped with Cu K_α_ X-ray radiation. The device was run at 40 kV and 40 mA. The basicity was determined by using the CO_2_ temperature-programmed desorption, where a sample of 0.1 g was washed in helium flow and was then saturated for 1.0 h at 200 °C in an atmosphere of 20 vol.% CO_2_/He. A carrier flow of helium at 25 mL/min was used in the temperature range of 25–720 °C (heating rate, 12 °C/min) for CO_2_ desorption. The morphology of the catalyst samples was examined via a high-resolution transmission electron microscope (HRTEM model: JEM-2100 F, JEOL, Akishima, Tokyo, Japan). The quantity of carbon deposit on the spent catalysts was determined by the thermo-gravimetric analysis. A platinum pan was filled with 10–15 mg of the spent catalyst. Heating was performed from room temperature to 900 °C at a rate of 20 °C min^−1^. The change in mass was constantly monitored as the heating progressed.

### 2.4. Catalyst Activity Test

The dry reforming of methane catalytic activity test started with a 0.1 g catalyst, put in stainless steel vertical fixed-bed tubular reactor (0.91 cm i.d. and 0.30 m long) (PID Eng. and Tech Micro Activity) by using a ball of glass wool. The reaction was carried out under 1.0 atm pressure. A K-type stainless sheathed thermocouple was used to maintain the temperature of the reaction. The catalyst was activated with reductive treatment under the flow of hydrogen (20 mL/min) for 60 min at 600 °C. Then, after eliminating the physiosorbed H_2_, the N_2_ treatment was performed for 15 min. The mixture of feed gas was CH_4_/CO_2_/N_2_ at 30, 30, 10 mL/min with a space velocity of 42,000 mL/(h.g_cat_)_,_ passed through the reactor. TCD-equipped gas chromatography (GC-2014 SHIMADZU, Kyoto, Japan) was used to analyse the product. The following expressions were used for the conversions:

(4) $${\mathrm{CH}}_{4}\;\mathrm{conversion}\;\left(\%\right)=\frac{{\mathrm{CH}}_{4,\mathrm{in}}-{\;\mathrm{CH}}_{4,\mathrm{out}\;}}{{\mathrm{CH}}_{4,\mathrm{in}}}\times 100$$

(5) $${\mathrm{CO}}_{2}\;\mathrm{conversion}\;\left(\%\right)=\frac{{\mathrm{CO}}_{2,\mathrm{in}}-{\;\mathrm{CO}}_{2,\mathrm{out}\;}}{{\mathrm{CO}}_{2,\mathrm{in}}}\times 100$$

## 3. Results

**Figure 1:**
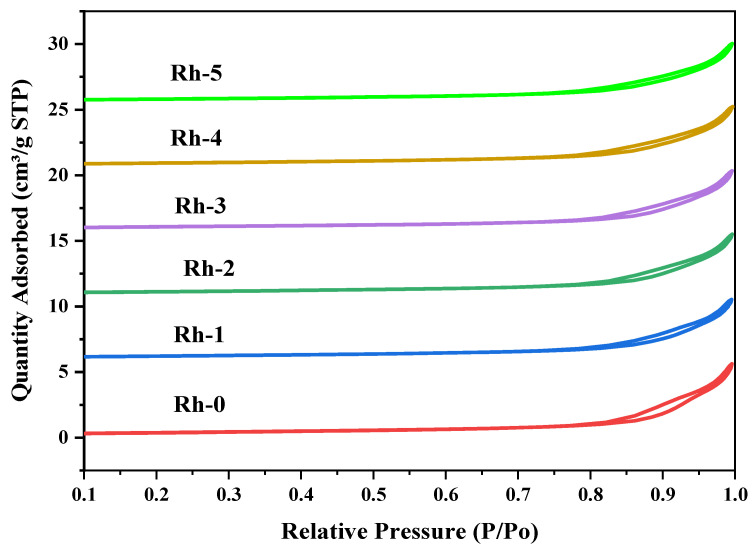
N_2_ physisorption of Rh-x (x = 0, 1, 2, 3, 4, and 5 wt.%).

The surface properties of the fresh catalysts were examined using nitrogen adsorption–desorption isotherms. Figure 1 shows the results of the isotherms. According to the IUPAC sorting system, the observed isotherms belonged to type IV classification with [38,39] an H3-type hysteresis loop, implying the existence of slit-like pores in the catalysts. In the range of 0.8–1.0, the relative pressure (P/P_0_) grew. In addition, the catalysts showed low specific volume adsorption of nitrogen gas, in the range of 4.5–8.0 cm^3^/g. Table 1 outlines the textural aspects of the samples. The catalysts showed specific surface areas in the range of 27–31 m^2^/g. Nevertheless, the pore diameter was in the range of 22–25 nm.

**Figure 2:**
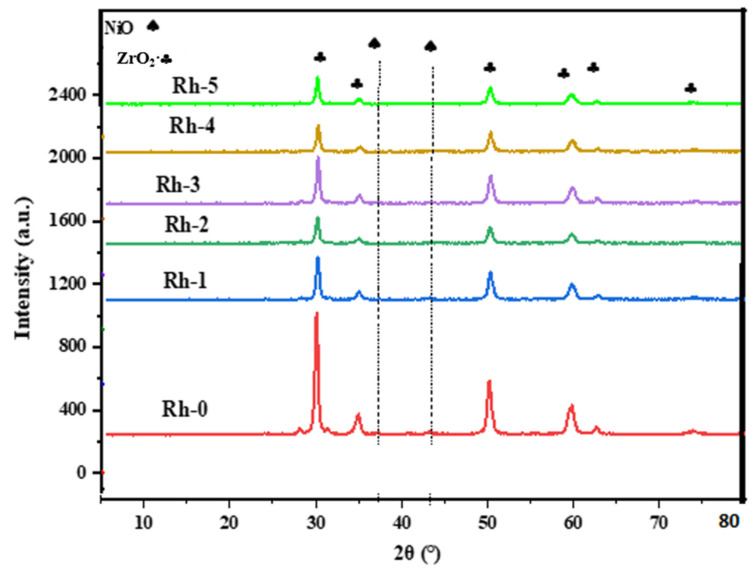
XRD pattern of fresh Rh-x (x = 0, 1, 2, 3, 4, and 5 wt.%) catalyst samples. (♠) NiO and (♣) cubic phases of yttria-stabilized zirconia samples.

Figure 2 exhibits the XRD patterns of the catalysts at 2θ° = 5–80°. The XRD pattern of yttria-stabilized zirconia support showed the cubic phase of zirconia (JCPDS No. 49-1642), which appeared at 2θ = 30°, 35°, 50°, 60°, 63°, and 75° in reference to the (111), (200), (220), (311), (222), and (400), respectively, in all catalysts. Meanwhile, the peaks for NiO were at 37.2° and 43.1°. The XRD patterns for the Rh_2_O_3_-promoted samples were identical to those of the unpromoted one, denoting that the introduction of Rh_2_O_3_ did not bring additional compounds. The absence of Rh_2_O_3_ in the XRD patterns indicated its fine dispersion on the surface.

**Figure 3:**
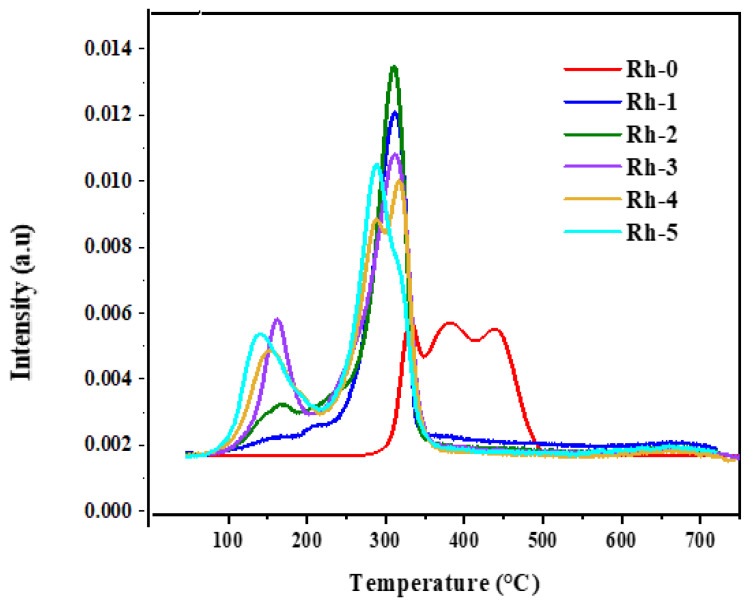
H_2_-TPR profiles of fresh Rh-x (x = 0, 1, 2, 3, 4, and 5 wt.%) catalysts.

To perform H_2_-temperature programming reduction (H_2_-TPR) analysis, a sample of ca. 100 mg of catalyst was placed in a r micro-reactor (i.d., 4 mm), fed with a 5 vol. % H_2_/Ar at a flow rate of 3.6 L/h at STP, in the range of 0–700 °C, with a heating rate of 12 °C/min. A thermal conductivity detector (TCD) monitored the hydrogen consumption. Figure 3 displays the TPR profiles of the fresh Rh-x (x = 0, 1, 2, 3, 4, and 5 wt.%) catalysts. The reduction peaks for the Rh-promoted Ni catalysts appeared in the low temperature range (80–250 °C). These were attributed to Ni catalysts loosely attached to the support, while the broad peaks with overlapped shoulders in the medium temperature range (300–500 °C) was for the unpromoted Ni catalysts, whereby the Ni was moderately attached to the support. The incorporation of the Rh-promoter caused the reduction peaks for nickel oxide to shift to lower temperatures in the range of 250–350 °C; the higher the loading of Rh was, the lower the reduction temperature was, viz. the weaker the interaction between the nickel oxide and the support was. Moreover, all the catalysts did not show any appreciable peak beyond 550 °C, stipulating that all the existing oxides were reduced and the absence of spinel phases like NiRh_2_O_3_. The incorporation of Rh_2_O_3_ (1–3 wt.%) slightly boosted the rate of H_2_ consumption, as shown in Table 2. Further increase in Rh_2_O_3_ loading (3–5 wt.%) did not affect the amount of hydrogen consumption, indicating that Rh_2_O_3_ was impeded under the surface of the catalysts.

**Figure 4:**
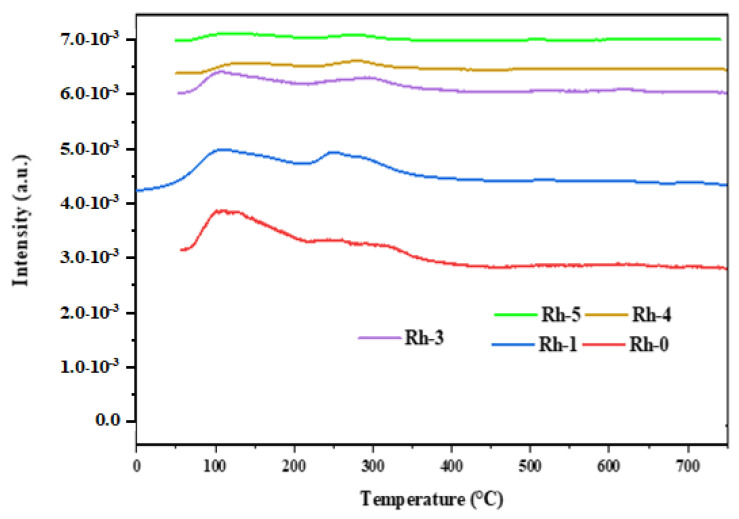
CO_2_-TPD profiles for Ni- Rh-x (x = 0, 1, 3, 4, 5 wt.%) catalysts.

The CO_2_-TPD characterization was conducted to justify the basicity effect that is observed by adding Rh_2_O_3_. Because CO_2_ adsorption capacity plays a fundamental role in promoting CO_2_ conversion in DMR, the effect of Rh_2_O_3_ on CO_2_ adsorption capacity was the first parameter to be considered [40]. Figure 4 demonstrates that all the catalysts did not effectively promote CO_2_ adsorption at temperatures above 400 °C. Thus, the low desorption temperatures of CO_2_ in the range of 50–400 °C reflected a weak basicity due to the availability of few basic sites on the surface of the catalysts [41,42]. The peak at 110 °C was ascribed to the adsorption of CO_2_ on OH groups which is weak, while peaks at 250 °C and 340 °C were attributed to moderate adsorption of CO_2_ on metal–oxygen pairs [43]. The increase in Rh_2_O_3_ amount produced peaks with lower intensities, and hence, further diminished the basicity of the catalysts. The obtained results underscored that the basic properties of the catalysts could be altered by doping the Rh_2_O_3_ promoter. Table 3 displays the amount of CO_2_ consumption in mol/g_cat_ during the CO_2_-TPD process. The Rh-promoted samples assumed values between 0.8 and 1.0.

**Figure 5:**
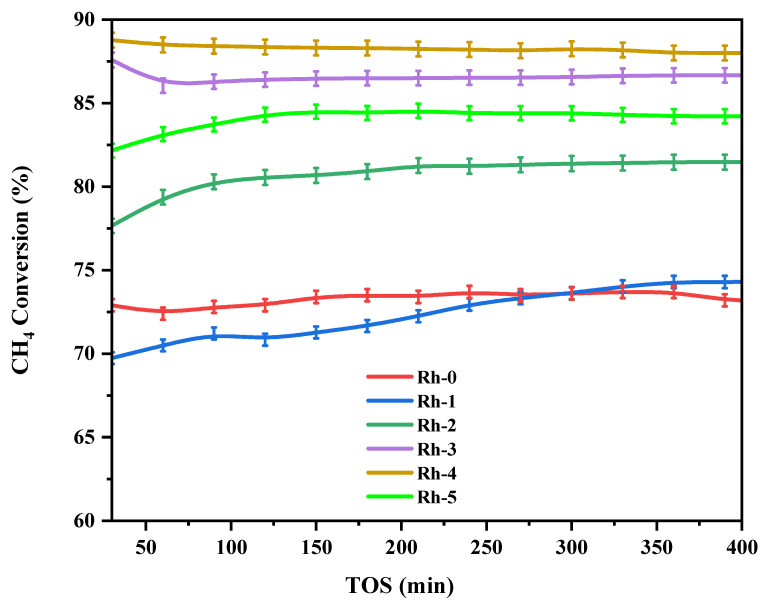
CH_4_ conversion versus TOS for the Ni-Rh-x (x = 0, 1, 2, 3, 4, 5 wt.%) catalysts operated at 800 °C.

**Figure 6:**
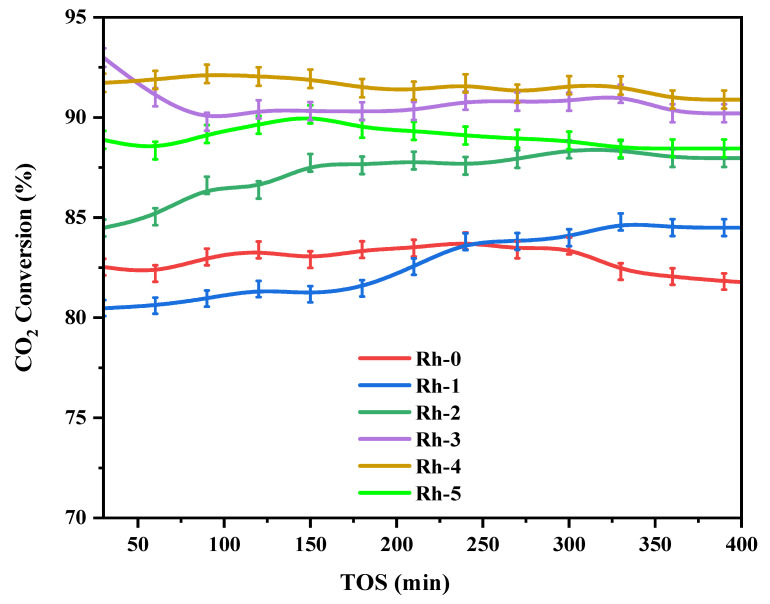
CO_2_ conversion versus TOS for the Ni-Rh-x (x = 0, 1, 2, 3, 4, 5 wt.%) catalysts operated at 800 °C.

Figure 5 displays the methane conversion profiles. The activity of the Ni-Rh-x (x = 0, 1, 2, 3, 4, 5 wt.%) catalysts was determined at 700 °C. The acquired activity clearly indicated that the addition of a Rh_2_O_3_ promoter had a profound impact on the performance. The various Rh_2_O_3_ loadings of the promoter increased the conversion values from 7 to 20% in comparison to the unpromoted catalyst. Indeed, the 4 wt.% Rh_2_O_3_ loadings gave the optimum activity value of 88% and the best stability since the activity drop was less than 1% during the 420 min of reaction. The improvement of conversion with Rh_2_O_3_ loadings up to 4 wt.% was credited to additional activity sites offered by the Rh metal. However, the higher loading of Rh_2_O_3_ such as 5 wt.% reduced the activity due to Rh covering the Ni active sites. Figure 6 shows the carbon dioxide conversion profiles. It also displayed a similar improvement in conversion with respect to the promotional effects of Rh_2_O_3_. The conversion values were higher than the corresponding CH_4_ conversion in Figure 5. This incident can be related to the occurrence of the reverse water gas shift reaction Equation (6), where the CO_2_ reacts with formed H_2_.

(6) $${\mathrm{CO}}_{2}+H_{2}\;↔H_{2}O+\mathrm{CO}$$

Moreover, it can be also attributed to the gasification of carbon deposition by carbon dioxide over metallic rhodium [41]. The catalytic efficiency of this research was compared to the results cited in the past in Table 4. The results show the relevance and the excellence of the adopted process for the formation of a competitive catalyst.

**Thermodynamic Analysis**

The Predictive Soave–Redlich–Kwong (PSRK) equation of state was used, as a thermodynamic model, to determine the changes in enthalpy (ΔH), entropy (ΔS), and Gibbs free energy (ΔG) at 800 °C for the main reaction of dry reforming of methane (DRM) and the side reaction of reverse water gas shift (RWGS) over each catalyst. The equilibrium constants (K_eq_) for these two reactions were calculated at 800 °C by using the “R_Equil_” model. All calculations were performed using Aspen Plus, V13. Table 5 shows the thermodynamic parameters and the equilibrium constants of the two reactions.

The equilibrium conversion of methane was found to be 73.6% at 800 °C. Because the produced hydrogen from DRM was simultaneously consumed by the RWGS, the equilibrium was shifted to higher values, as stated by Le Chatelier’s principle.

As shown in Table 5, the values of ΔH, ΔS, and ΔG increased with increasing Rh_2_O_3_ content (the promoter) from 0.0 to 4.0 wt.%. Such observations indicated that less carbon dioxide and hydrogen were involved in RWGS with increased loading of the promoter due to the much higher endothermic nature of DRM (ΔH^°^_298_ = 247,021 kJ/kmol) than that of RWGS (ΔH^°^_298_ = 41,058 kJ/kmol) and the much higher K_eq_ value of DRM than that of RWGS. However, increasing the promoter content to 5.0 wt.% led to a reduction in the values of these thermodynamic parameters, probably owing to the coverage of some nickel metal active sites by some rhodium metal particles, and hence, the loss of some of the synergetic effect between the nickel and rhodium metals. Therefore, this thermodynamics analysis provided a support for the highest observed catalytic performance for the Rh-4 catalyst.

**Figure 7:**
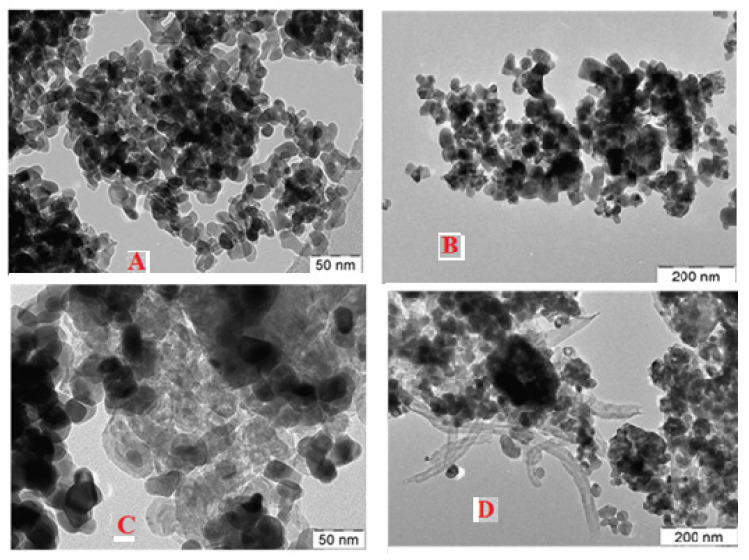
TEM images of (**A**) fresh Rh-0; (**B**) fresh Rh-4; (**C**) spent Rh-0; (**D**) spent Rh-4.

Figure 7 displays the TEM images of the Ni-Rh-x (x = 0, 4 wt.%) catalysts at 50 or 200 nm scales. Figure 7A,B show fresh Rh-0 and Rh-4, respectively, where the agglomerated catalysts particles showed the same morphology irrespective of Rh_2_O_3_ promoter loading. Figure 7C,D show spent Rh-0 and Rh-4, respectively. The spent catalyst particles showed the formation of filamentous carbon.

**Figure 8:**
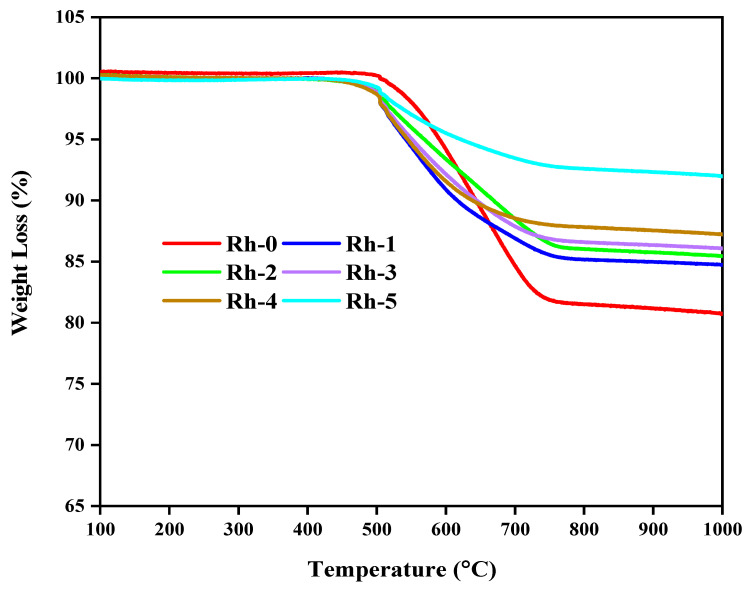
TGA profiles for the Rh-x (x = 0, 1, 2, 3, 4, 5 wt.%) catalysts after TOS of 420 min in DRM, at 800 °C and 1 atm.

For the quantitative determination of carbon formations after the reaction, TGAs of the Ni-Rh-x (x = 0, 1, 2, 3, 4, 5 wt.%) catalysts were performed under an air atmosphere (Figure 8). The un-promoted catalyst Rh-0 gave the highest amount of weight loss (18.5%). The gradual addition of Rh_2_O_3_ clearly influenced the coke formation, where 7–15 wt.% of coke was deposited on catalysts containing 1–5 wt.% Rh_2_O_3_. The optimally promoted catalyst (Rh-4) provided the least weight loss, with the exclusion of Rh-5, which was marked by a reaction drop. The observed trend in the reduction of the amount of carbon deposition with increasing Rh_2_O_3_ loading could be ascribed to the ability of Rh to catalyse carbon oxidation via carbon gasification by carbon dioxide and steam [41], as shown in the following chemical equations:

(7) $${\mathrm{CO}}_{2}+C\;↔2\mathrm{CO}$$

(8) $$H_{2}O+C\;↔\mathrm{CO}+H_{2}$$

On this basis, the carbon formation over our catalysts in the process of DRM might be attributed to the decomposition of methane [52]:

(9) $${\mathrm{CH}}_{4}↔2H_{2}+C$$

## 4. Conclusions

In this article, the impact of a rhodium oxide promoter on a nickel catalyst supported over yttria-stabilized zirconia for CH_4_ reforming using a CO_2_ oxidant was investigated at 800 °C. The dry impregnation method was used for the synthesis of all catalysts. The characterization results substantiated that the addition of Rh_2_O_3_ did not markedly affect the morphology. However, it lowered the basicity, reduced the coke formation, and brought down the reduction temperature. These combined qualities demonstrated that 4 wt.% loading of Rh_2_O_3_ gave the optimum catalyst performance in activity and stability during the dry reforming of methane with minimization of reverse water gas shift, as inferred from the thermodynamics analysis.

## Figures and Tables

**Table 1:** Textural aspects of the catalysts.

Sample-Type	BET Area (m^2^/g)	Pore Volume (cm^3^/g)	Pore Diameter (nm)
Rh-0	31	0.19	25.00
Rh-1	27	0.15	23.00
Rh-2	27	0.16	23.00
Rh-3	27	0.15	24.00
Rh-4	28	0.15	22.00
Rh-5	27	0.15	22.00

**Table 2:** H_2_ consumption for promoted and unpromoted Rh_2_O_3_ catalysts.

Sample	Description	H_2_ Consumption (mol/g_cat_)
Rh-0	5% Ni /8%Y_2_O_3_ + 92% ZrO_2_	0.68
Rh-1	5% Ni+ 1% Rh/8% Y_2_O_3_ + 92% ZrO_2_	0.80
Rh-2	5% Ni+ 2% Rh/8% Y_2_O_3_ + 92% ZrO_2_	0.90
Rh-3	5% Ni+ 3% Rh/8% Y_2_O_3_ + 92% ZrO_2_	1.00
Rh-4	5% Ni+ 4% Rh/8% Y_2_O_3_ + 92% ZrO_2_	1.00
Rh-5	5% Ni+ 5% Rh/8% Y_2_O_3_ + 92% ZrO_2_	1.00

**Table 3:** H_2_ consumption for promoted Rh_2_O_3_ catalysts.

Sample	Description	CO_2_ Consumption (μmol/g_cat_)
Rh-0	5% Ni /8%Y_2_O_3_ + 92% ZrO_2_	0.72
Rh-1	5% Ni+ 1% Rh/8% Y_2_O_3_ + 92% ZrO_2_	0.8
Rh-2	5% Ni+ 2% Rh/8% Y_2_O_3_ + 92% ZrO_2_	0.9
Rh-3	5% Ni+ 3% Rh/8% Y_2_O_3_ + 92% ZrO_2_	1.0
Rh-4	5% Ni+ 4% Rh/8% Y_2_O_3_ + 92% ZrO_2_	1.0
Rh-5	5% Ni+ 5% Rh/8% Y_2_O_3_ + 92% ZrO_2_	1.0

**Table 4:** Catalytic performance of new method compared to previous works for the dry reforming of methane.

Catalyst	T (°C)	CH_4_/CO_2_	CH_4_ Conversion (%)	Ref.
Ni-2.5%Ce/W-Zr	700	1:1	78	[44]
Ni-CeO_2_@SiO_2_	800	1:1	80	[45]
Co-Ni/Sc-SBA-15	700	1:1	66	[46]
Ni@SiO_2_	700	1:1	71	[47]
Ni-Co/ZrO_2_-CaO + SiC	800	1:1	97	[48]
5.6 wt.% Ni/Al_2_O_3_ + FY5	800	1:1	90	[49]
10%Ni/ Al_2_O_3_ − F	700	1:1	72	[50]
20%Ni/ Al_2_O_3_ (mixed with biochar)	800	1:1	79	[51]
5% Ni+ 4% Rh/8% Y_2_O_3_ + 92% ZrO_2_	800	1:1	89	The present work

**Table 5:** Calculated thermodynamic parameter over the various catalysts at 800 °C.

Catalyst	Thermodynamics Parameter		
	ΔH_800_, kJ/kmol	ΔS_800_, kJ/(kmol.K)	ΔG_800_, kJ/kmol
Rh-0	+136,845.2	+54.4208	+78,444
Rh-1	+137,654.4	+54.5777	+79,085
Rh-2	+145,049.0	+56.2075	+84,730
Rh-3	+150,385.0	+57.2514	+88,946
Rh-4	+151,860.1	+57.5033	+90,151
Rh-5	+148,103.0	+56.8009	+87,147
**Reaction**			**K_eq_ (800 °C)**
DRM: CH_4(g)_ + CO_2(g)_ = 2H_2(g)_ + 2CO_(g)_			151.348
RWGS: CO_2(g)_ + H_2(g)_ = CO_(g)_ + H_2_O_(g)_			0.912605
